# Sulfur-Doped Graphene-Based Electrochemical Sensors for Fast and Sensitive Determination of (R)-(+)-Limonene from Beverages

**DOI:** 10.3390/s22155851

**Published:** 2022-08-05

**Authors:** Andreea-Roxana Niculae, Raluca-Ioana Stefan-van Staden, Jacobus Frederick van Staden, Ramona Georgescu State

**Affiliations:** 1 Laboratory of Electrochemistry and PATLAB, National Institute of Research for Electrochemistry and Condensed Matter, 202 Splaiul Independentei Str., 060021 Bucharest, Romania; 2 Faculty of Chemical Engineering and Biotechnologies, Politehnica University of Bucharest, 060021 Bucharest, Romania

**Keywords:** limonene, differential pulse voltammetry, electrochemical sensor, food samples

## Abstract

Two sensors based on sulfur-doped graphene, a gold nanoparticle paste modified with 5,10,15,20-tetraphenyl-21H,23H-porphine and 5,10,15,20-tetrakis (pentafluorophenyl chloride)-21H,23H-iron (III) porphyrin, were proposed for the determination of R-limonene in beverages (triple sec liqueur and limoncello). Differential pulse voltammetry was the method used to characterize and validate the proposed sensors. The response characteristics showed that the detection limits for both sensors were 3 × 10^−6^ mol L^−1^, while the quantification limits were 1 × 10^−5^ mol L^−1^. Both sensors can be used to determine R-limonene in a concentration range between 1 × 10^−5^–6 × 10^−4^ mol L^−1^ for TPP/AuNPs-S-Gr and 1 × 10^−5^–1 × 10^−3^ mol L^−1^ for Fe(TPFPP)Cl/AuNPs-S-Gr. The highest sensitivity (0.7068 µA/mol L^−1^) was recorded when the TPP/AuNPs-S-Gr sensor was used, proving that the electrocatalytic effect of this electrocatalyst is higher compared to that of Fe(TPFPP)Cl/AuNPs-S-Gr. High recoveries (values greater than 99.00%) and low RSD values (%) (below 5.00%) were recorded for both sensors when used to determine R-limonene in triple sec liqueur and limoncello.

## 1. Introduction

(R)-(+)-limonene (R-LIM) is a monoterpene present in essential oils from the citrus peel. This can be extracted from the essential oil by distillation at low pressure. Improper storage will cause this refined (R)-(+)-limonene to quickly oxidize in the air, resulting in a lower purity of 95–96% [1,2].

Many textbooks of organic chemistry [3] still attribute (S)-(−)-limonene to the smell of lemons and (R)-(+)-limonene to that of oranges. As a result, many chemists share this belief, including the authors of a research study on optical rotation in which essential oils were examined. They found that lemon oil rotates clockwise, but to a lesser extent than orange oil [4]. This finding led to an investigation that included odor tests, chiral and achiral GC analyses, and a search for the source of the claim that (S)-(−)-limonene, which is the dominant component in lemon oil (more than 98 to percent), is the trigger for the smell of lemon and that (R)-(+)-limonene, which is the dominant ingredient in orange oil (more than 98 percent), is the trigger for the smell of oranges. Because of its position as an air pollutant and its link to negative health impacts, limonene is therefore interesting. R-limonene and S-limonene are the two enantiomers of limonene, which has a citrus or orange fragrance. Oranges are a common example of a biosynthesized source of limonene, and it can also be chemically produced for use in scent formulations [5,6].

For limonene determination, many analytical methods, such as gas chromatography–mass spectrometry (GS–MS) [7], high-performance liquid chromatography/tandem mass spectrometry (HPLC/MS) [8], and ultra performance liquid chromatography (UPLC) [9], were reported. Although these methods are widely used to determine limonene from various samples, they have disadvantages such as expensive equipment, a long compound analysis time, large quantities of solvents and laborious stages of sample preparation. Taking into account these less advantageous aspects of classical methods of determination, electrochemical methods have advantages such as reduced costs for the equipment, reduced processing time, fast response and reduced amount of solvents and samples.

By doping graphene with heteroatoms such as F, S, B, and N, an improvement in electroconductivity and dispersibility was observed [10,11,12,13,14,15,16,17,18,19]. In this paper, the doping of graphene with S (S-Gr) was chosen for its effects on the π electrons in the carbon lattice, namely the modification of the structure of graphene; higher sensitivities are expected when used for sensor design [20].

Gold nanoparticles (AuNPs) have also been used to maximize the electroactive surface area of the sensor, facilitating electronic transfer. At the same time, they were chosen for their good conductivity, sensitivity and the improved transfer of the reactant mass [10]. Porphyrins and metalloporphyrins are well known for their catalytic properties for chemical reactions, especially reactions involving electron transfer [21,22].

In this work, two electrochemical sensors based on sulfur-doped graphene (S-Gr), a gold nanoparticle paste modified with 5,10,15,20-tetraphenyl-21H,23H-porphine (TPP) and 5,10,15,20-tetrakis(pentafluorophenyl)-21H,23H-porphyrin iron(III) chloride [Fe(TPFPP)Cl] were designed, characterized, and validated for determination of R-limonene in beverages. Selection of an iron complex of porphyrin versus a porphyrin was to check the effect of iron complex on the electrocatalytic oxidation of limonene versus the electrocatalytic oxidation effect of TPP on limonene.

## 2. Materials and Methods

R-(+)-Limonene, sulfur-doped graphene, gold nanoparticles (10 nm diameter, OD 1, stabilized suspension in 0.1 mmol L^−1^ PBS, reactant free), 5,10,15,20-tetraphenyl-21H,23H-porphine, 5,10,15,20-tetrakis(pentafluorophenyl)-21H,23H-porphyrin iron(III) chloride, sucrose, D-glucose, L-ascorbic acid, magnesium chloride, potassium chloride, iron(III) nitrate, and sodium chloride were bought from Sigma Aldrich, and paraffin oil (d420, 0.86 g cm^−1^) was purchased from Fluka (Buchs, Sweden). The sulfur-doped graphene powder was synthesized in house, and characterized using SEM and other structural methods of analysis as described earlier [10].

The phosphate buffer solution (PBS, 0.1 mol L^−1^) was prepared by mixing sodium phosphate monobasic monohydrate and sodium phosphate dibasic heptahydrate solutions. The pH of the buffer solution was adjusted to pH 1.5, 1.8, 2.0, 3.0, 4.0, 5.0 and 6.0 using HCl and NaOH solutions.

The stock solution of R-limonene (1 × 10^−2^ mol L^−1^) was prepared in ethanol. Before the measurements, the working solutions were freshly prepared and protected from sunlight.

The mini potentiostat EmSTAT Pico (software PsTrace 5.8 PalmSens, Houten, The Netherlands) linked to a laptop for data acquisition was used for all measurements: cyclic voltammetry (CV), differential pulse voltammetry (DPV) and electrochemical impendence spectroscopy (EIS) measurements. All electrochemical experiments were realized at 25 °C. The results were recorded using an electrochemical cell containing three electrodes: working electrode—the modified sulfur-doped graphene and gold nanoparticle paste electrode, Ag/AgCl (0.1 mol L^−1^ KCl) and Pt-wire as working, reference and auxiliary electrodes, respectively. The pH adjustment was performed using a Mettler Toledo pH meter.

An amount of 100 mg sulfur-doped graphene powder was mixed with 10 µL gold nanoparticle suspension and paraffin oil was added until a homogeneous paste was obtained. The modified electrochemical sensors were prepared by addition of 25 µL from a solution of 5,10,15,20-tetraphenyl-21H,23H-porphine (1 × 10^−3^ mol L^−1^ in tetrahydrofuran) and 25 µL from a solution of 5,10,15,20-tetrakis(pentafluorophenyl)-21H,23H-porphyrin iron(III) chloride (1 × 10^−3^ mol L^−1^ in tetrahydrofuran) to the bare paste. The TPP/AuNPs-S-Gr and Fe(TPFPP)Cl/AuNPs-S-Gr electrodes were obtained. The pastes were placed into non-conducting plastic tubes (internal diameter 25 μm) in which a silver wire inserted into the paste served as electrical contact between the paste and external circuit.

The new developed sensors were used to detect R-limonene in two beverages: limoncello and triple sec liqueur, bought from a Romanian supermarket. The samples were diluted in PBS pH 2.0 and pH 3.0, respectively, in a 1:1 (*v*/*v*) ratio and afterward spiked with different concentrations of R-LIM.

## 3. Results and Discussion

### 3.1. Electrochemical Characterization of the Sensor

The electrochemical characterization of the TPP/AuNPs-S-Gr and Fe(TPFPP)Cl/AuNPs-S-Gr sensors compared to the sensor based on sulfur-doped graphene and AuNPs, was done using: differential plus voltammetry (DPV), spectroscopy electrochemical impedance (EIS) and cyclic voltammetry.

**Figure 1:**
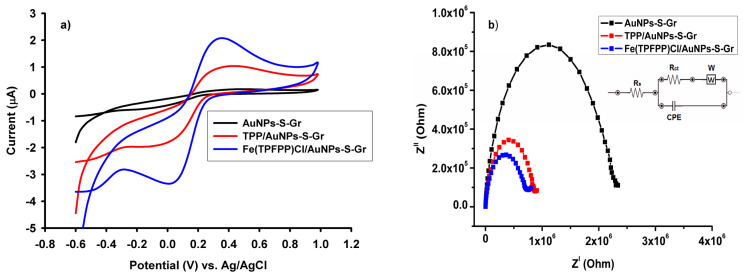

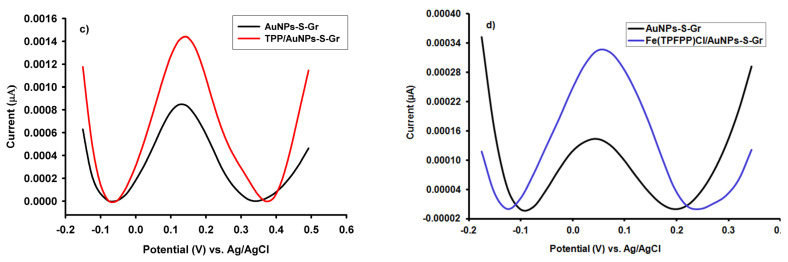
(**a**) Cyclic voltammograms of the current plotted against the potential (working conditions: step potential 0.025 V; scan rate 0.1 V s^−1^) in a solution of 5.0 × 10^−3^ mol L^−1^ K_3_[Fe(CN)_6_] (0.1 mol L^−1^ KCl) using the AuNPs-S-Gr (black line), TPP/AuNPs-S-Gr (red line) and Fe(TPFPP)Cl/AuNPs-S-Gr (blue line). (**b**) Electrochemical impedance spectra recorded for AuNPs-S-Gr (black line), TPP/AuNPs-S-Gr (red line) and Fe(TPFPP)Cl/AuNPs-S-Gr (blue line) in a solution of 5.0 × 10^−3^ mol L^−1^ K_3_[Fe(CN)_6_] (0.1 mol L^−1^ KCl) (conditions: frequency range between 1.0 × 10^5^ to 1.0 × 10^−1^ Hz). Inset: equivalent circuit diagram of the electrochemical interface used to fit the impedance spectra where R_s_ is the solution resistance, CPE is the constant phase element, R_ct_ is the electron-transfer resistance, and W is the Warburg diffusion resistance. (**c**) The recorded differential pulse voltammograms in PBS pH 2.0 containing 1 × 10^−4^ mol L^−1^ R-limonene for the AuNPs-S-Gr (black line) and TPP/AuNPs-S-Gr (red line). (**d**) The recorded differential pulse voltammograms in PBS pH 3.0 containing 1 × 10^−4^ mol L^−1^ R-limonene for the AuNPs-S-Gr (black line) and Fe(TPFPP)Cl/AuNPs-S-Gr (blue line).

The electrochemical response of the modified sensors was analyzed using the CV method (Figure 1a). Cyclic voltammograms were obtained for a solution of 5.0 × 10^−3^ mol L^−1^ K_3_[Fe(CN)_6_] (0.1 mol L^−1^ KCl) in a potential range between −0.6 V and 1.0 V, using as working electrodes, AuNPs-S-Gr, TPP/AuNPs-S-Gr and Fe(TPFPP)Cl/AuNPs-S-Gr (Figure 1a). It can be seen that after modifying the AuNPs-S-Gr paste with TPP and Fe(TPFPP)Cl the conductivity of the sensors increased. Therefore, by modifying the sensors, the electrochemical response was improved.

The electrode interface was studied using the EIS method in the frequency range from 1.0 × 10^5^ to 1.0 × 10^−1^ Hz in a solution of 5.0 × 10^−3^ mol L^−1^ K_3_[Fe(CN)_6_] (0.1 mol L^−1^ KCl). Nyquist diagrams are shown in Figure 1b. In Figure 1b is observed that the largest semicircle belongs to the unmodified sensor, with Rct = 1.66 × 10^5^ Ω, the smallest semicircle belongs to the TPP/AuNPs-S-Gr sensor (Rct = 1.80 × 10^5^ Ω), while for Fe(TPFPP)Cl/AuNPs-S-Gr sensor, the semicircle diameter decreased (Rct = 8.14 × 10^4^ Ω). Therefore, the Fe(TPFPP)Cl/AuNPs-S-Gr sensor showed a smaller semicircle and a highest Rct value than the Rct values obtained for the TPP/AuNPs-S-Gr and AuNPs-S-Gr sensors.

The electrochemical behavior of TPP/AuNPs-S-Gr and Fe(TPFPP)Cl/AuNPs-S-Gr, was compared with AuNPs-S-Gr, using the DPV method, in a solution containing 1 × 10^−4^ mol L^−1^ R-LIM buffered with PBS at pH 2.0 and pH 3.0, respectively. The voltammograms recorded for the sensors are illustrated in Figure 1c,d. Comparing the three sensors (Figure 1c,d), the TPP/AuNPs-S-Gr and Fe(TPFPP)Cl/AuNPs-S-Gr gave the best results for R-LIM oxidation.

TPP/AuNPs-S-Gr worked much better than the sensor Fe(TPFPP)Cl/AuNPs-S-Gr. Therefore, the two sensors were further characterized and tested for electrochemical determination of R-LIM in alcoholic beverages samples such as limoncello and triple sec liqueur.

**Figure 2:**
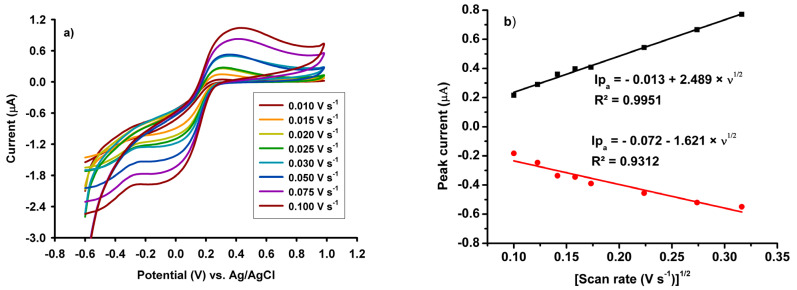
(**a**) Cyclic voltammograms in a solution of 5.0 × 10^−3^ mol L^−1^ K_3_[Fe(CN)_6_] (0.1 mol L^−1^ KCl) at different scan rates from 0.010 to 0.100 V s^−1^ using the TPP/AuNPs-S-Gr sensor (working conditions: potential range from −0.6 to 1.0 V; step potential 0.025 V). (**b**) Dependence of the peak current on the square root of the scan rate, using the TPP/AuNPs-S-Gr sensor.

**Figure 3:**
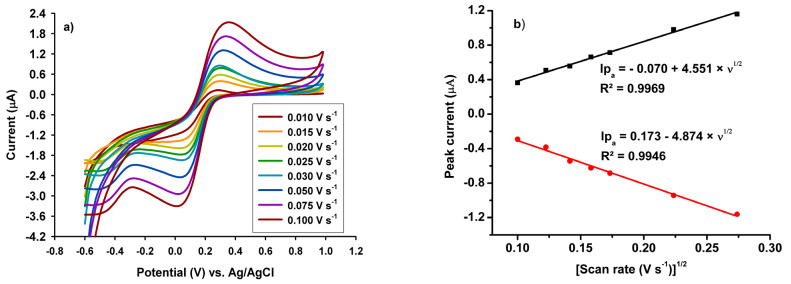
(**a**) Cyclic voltammograms in a solution of 5.0 × 10^−3^ mol L^−1^ K_3_[Fe(CN)_6_] (0.1 mol L^−1^ KCl) at different scan rates from 0.010 to 0.100 V s^−1^ using the Fe(TPFPP)Cl/AuNPs-S-Gr sensor (working conditions: potential range from −0.6 to 1.0 V; step potential 0.025 V). (**b**) Dependence of the peak current on the square root of the scan rate, using the Fe(TPFPP)Cl/AuNPs-S-Gr sensor.

The Randles–Sevcik equation was used to evaluate the electrocatalytic activity of the three sensors, when the electroactive surface area was calculated. For this purpose, a solution of 5.0 × 10^−3^ mol L^−1^ K_3_[Fe(CN)_6_] (0.1 mol L^−1^ KCl) was used. The variation of the scanning rate from 0.010 to 0.100 V s^−1^, showed that the anodic and cathodic peaks (Ip_a_ and Ip_c_) are in a linear dependence on the square root of the scanning rate, suggesting a diffusion-controlled redox process. In Figure 2a and Figure 3a, the increase in the scanning rate with the current intensity is shown. Figure 2b and Figure 3b show the linear dependencies for the Ip_a_ and Ip_c_ peaks vs. ν^1/2^. The active areas for TPP/AuNPs-S-Gr and Fe(TPFPP)Cl/AuNPs-S-Gr were 7 × 10^−4^ and 12 × 10^−4^ cm^2^, respectively, these values being higher than that obtained for the AuNPs-S-Gr sensor (7 × 10^−5^ cm^2^).

### 3.2. The Influence of the pH Value on the Electrochemical Behavior of the Sensors

**Figure 4:**
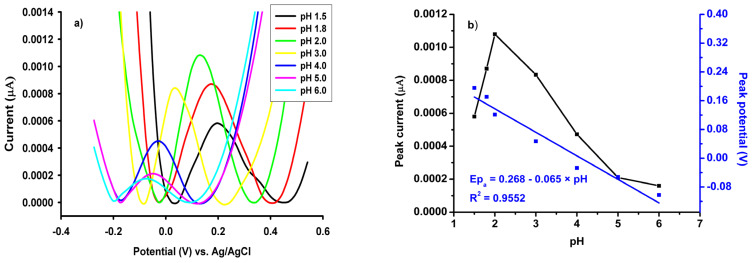
(**a**) Differential pulse voltammograms of 1 × 10^−4^ mol L^−1^ R-limonene using the TPP/AuNPs-S-Gr sensor in PBS at pH values of 1.5, 1.8, 2.0, 3.0, 4.0, 5.0, and 6.0. (**b**) The effect of the pH on the peak current (black dots) and the linear dependence of the peak potential with the pH (blue line).

The pH value of the solution is important in electrochemical measurements to determine the influence of the pH value on the R-LIM analysis. The R-LIM solution with a concentration of 100 µmol L^−1^ was buffered with PBS at different pH values (1,5, 1,8, 2,0, 3,0, 4,0, 5,0, and 6.0). Figure 4 shows that for the TPP/AuNPs-S-Gr sensor, the intensity of the current increased from pH 1.5, registering the maximum value of the current at pH 2.0, following a decrease in the intensity current, from pH 2.0 to pH 6.0 (Figure 4b). Figure 4b shows that at pH 2.0, the highest current intensity was reached for R-LIM analysis.

**Figure 5:**
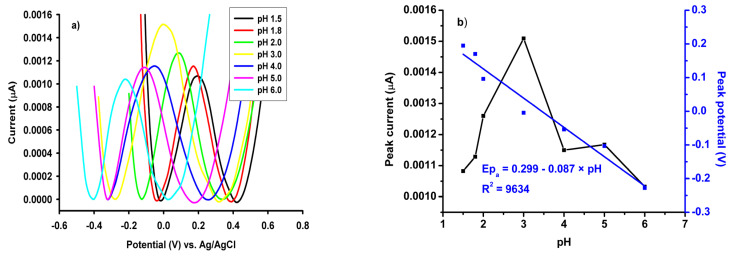
(**a**) Differential pulse voltammograms of 1 × 10^−4^ mol L^−1^ R-limonene using the Fe(TPFPP)Cl/AuNPs-S-Gr sensor in PBS at pH values of 1.5, 1.8, 2.0, 3.0, 4.0, 5.0, and 6.0. (**b**) The effect of the pH on the peak current (black dots) and the linear dependence of the peak potential with the pH (blue line).

In the case of the Fe(TPFPP)Cl/AuNPs-S-Gr sensor, the peak current increased from pH 1.5 to pH 3.0, which is the maximum value (Figure 5). Then, a decrease in pH to 6.0 was recorded (Figure 5).

### 3.3. Response Characteristics of the Electrochemical Sensors

The response characteristics of the modified electrochemical sensors were determined by differential pulse voltammetry, at the optimum pH of 2.0 for the TPP/AuNPs-S-Gr sensor and pH of 3.0 for the Fe(TPFPP)Cl/AuNPs-S-Gr sensor. The results obtained are presented in Table 1.

**Figure 6:**
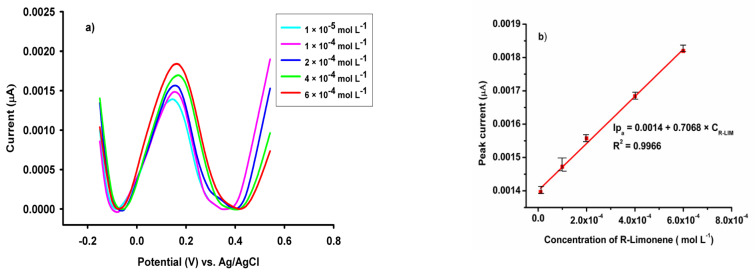
(**a**) Differential pulse voltammograms recorded with the TPP/AuNPs-S-Gr sensor in PBS pH 2.0 containing different concentrations of R-limonene from 1 × 10^−5^ mol L^−1^ to 6 × 10^−4^ mol L^−1^. (**b**) Calibration curves obtained with the TPP/AuNPs-S-Gr sensor from 1 × 10^−5^ mol L^−1^ to 6 × 10^−4^ mol L^−1^. Working conditions: step potential of 0.025 V; scan rate of 0.025 V s^−1^.

The dependence between the peak current and the concentration of R-LIM was linear (Figure 6b). Figure 6a showed for the TPP/AuNPs-S-Gr sensor, the peaks obtained during calibration, as well as the calibration graph for R-LIM. The linear concentration range was between 1 × 10^−5^ and 6 × 10^−4^ mol L^−1^ with a good regression coefficient.

The limit of detection (LOD) was 3 × 10^−6^ mol L^−1^ and the limit of quantification (LOQ) was 1 × 10^−5^. LOD and LOQ were calculated as follows: LOD = 3 s/m, LOQ = 10 s/m; where s—standard deviation of the peak current of the blank (4 measurements) and m—the slope of the calibration curve. The sensitivity of the TPP/AuNPs-S-Gr sensor was 0.7068 µA/mol L^−1^.

**Figure 7:**
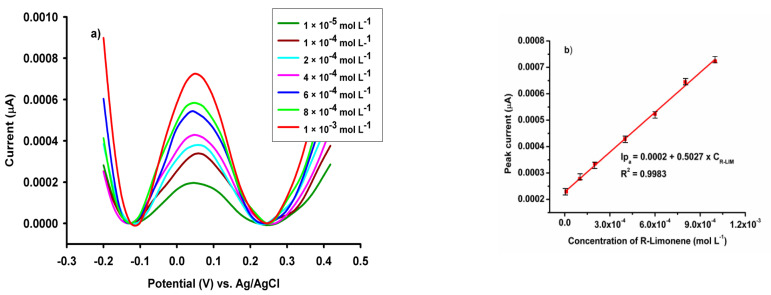
(**a**) Differential pulse voltammograms recorded with the Fe(TPFPP)Cl/AuNPs-S-Gr sensor in PBS pH 3.0 containing different concentrations of R-limonene from 1 × 10^−5^ mol L^−1^ to 1 × 10^−3^ mol L^−1^. (**b**) Calibration curves obtained with the Fe(TPFPP)Cl/AuNPs-S-Gr sensor from 1 × 10^−5^ mol L^−1^ to 1 × 10^−3^ mol L^−1^. Working conditions: step potential of 0.025 V; scan rate of 0.025 V s^−1^.

For the Fe(TPFPP)Cl/AuNPs-S-Gr sensor, Figure 7a showed the peaks obtained during calibration, as well as the calibration graph for R-LIM. The linear concentration range was between 1 × 10^−5^ and 1 × 10^−3^ mol L^−1^. The LOD and LOQ were calculated to be 3 × 10^−6^ and 1 × 10^−5^ mol L^−1^, respectively. These were calculated using the above formulas. The sensitivity of the Fe(TPFPP)Cl/AuNPs-S-Gr sensor was 0.5027 µA/mol L^−1^.

**Scheme 1:**
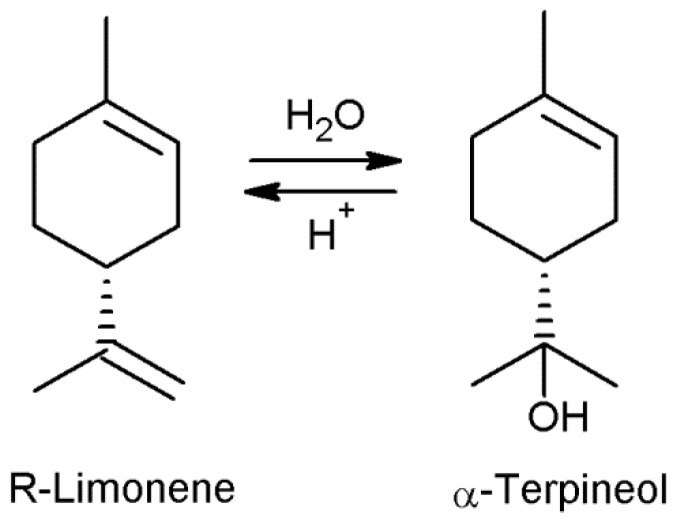
Proposed electrooxidation mechanism of R(+)-limonene on the proposed sensors.

The possible electrochemical oxidation mechanism of R(+)-limonene was presented in Scheme 1. The compound generated during the oxidation of R-limonene is α-terpineol, as the R-limonene molecule interacts with one water molecule. The carbenium ion was created as a result of the isomerization of R-limonene during oxidation. The α-terpineol compound was resulted by the acid’s nucleophilic attack on R-limonene [23].

### 3.4. Selectivity of the Electrochemical Sensor

Mg^2+^, K^+^, Na^+^, Fe^3+^ and organic species such as S-limonene, ascorbic acid, glucose, sucrose were tested as possible interferences in the determination of R-limonene. Possible interfering substances were chosen from the list of substances in beverages such as limoncello and triple sec liqueur. The enantioselectivity was tested versus S-limonene. The tolerance limit was defined as the maximum interference concentration that caused a change in current intensity in terms of relative error (±5% acceptance level) and Bias (%). All evaluated solutions were obtained under optimal conditions, pH 2.0 PBS with a constant concentration of R-LIM (1 × 10^−4^ mol L^−1^) for TPP/AuNPs-S-Gr and pH 3.0 with a constant concentration of R-LIM for Fe(TPFPP)Cl/AuNPs-S-Gr. The experimental results (Table 2 and Table 3) did not show any obvious influence on the detection of R-LIM when an excess of 10-fold of Mg^2+^, K^+^, Na^+^, Fe^3+^, ascorbic acid, glucose and sucrose was added.

The results presented in Table 2 and Table 3 also proved the enantioselectivity of the proposed sensors when used for the assay of R(+)-limonene. The enantioselectivity can be explained as follows: porphyrins and complexes of metals (including Fe(III)) with porphyrins played the role of an artificial enzyme (as they mimics the enzymes) by favorizing only the oxidation of R(+)-limonene, and not of S(−)-limonene; therefore, the sensors are enantioselective. In this case, it is not a substrate-binding process, but a electrocatalysis process in which the role of the electrocatalyst is taken by the artificial enzymes: TPP and Fe(TPFPP)Cl. The porphyrins mimic enzymes; the role of porphyrins as artificial enzymes in electrochemistry was extensively studied before [24,25,26,27].

### 3.5. Reproducibility, Repeatability and Stability

**Figure 8:**
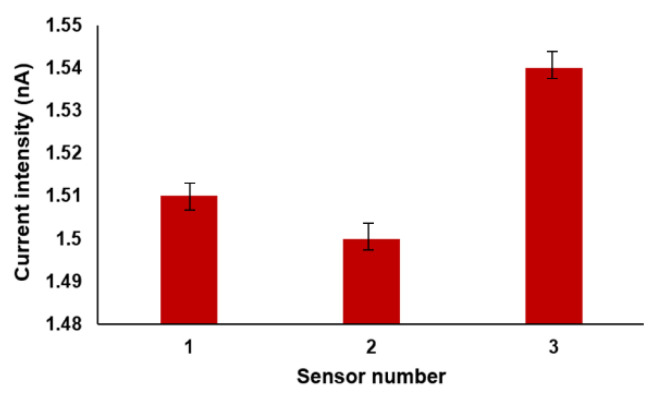
Reproducibility of the TPP/AuNPs-S-Gr sensor.

**Figure 9:**
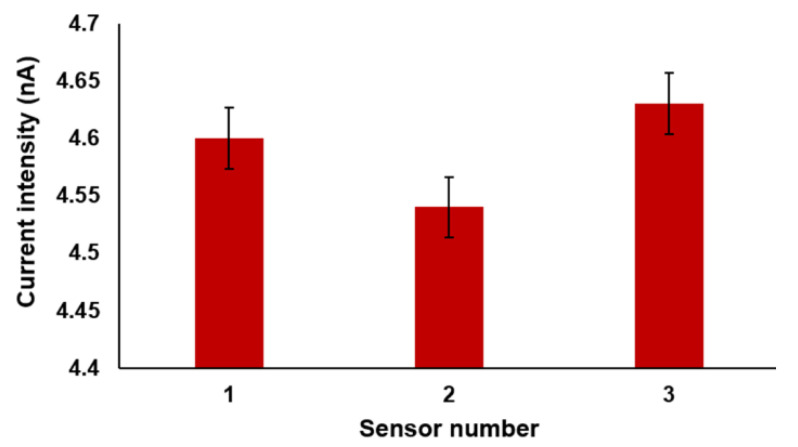
Reproducibility of the Fe(TPFPP)Cl/AuNPs-S-Gr sensor.

The repeatability, reproducibility and stability of the developed sensors (TPP/AuNPs-S-Gr and Fe(TPFPP)Cl/AuNPs-S-Gr) were investigated using a solution of R-limonene (1 × 10^−4^ mol L^−1^) in PBS pH 2.0 and 3.0, respectively, under the optimal experimental conditions by DPV. The reproducibility was analyzed using three new sensors of each type, which were prepared in the same way. The relative standard deviation (RSD%) was calculated to be 1.31% (n = 5) for the TPP/AuNPs-S-Gr sensor and 1.41% (n = 5) for the Fe(TPFPP)Cl/AuNPs-S-Gr sensor. For the within-day repeatability, the repeatability was determined to be 1.97% for the TPP/AuNPs-S-Gr sensor and 2.09% for the Fe(TPFPP)Cl/AuNPs-S-Gr sensor. These values of RSD% were obtained for five repetitive measurements (Figure 8 and Figure 9).

**Figure 10:**
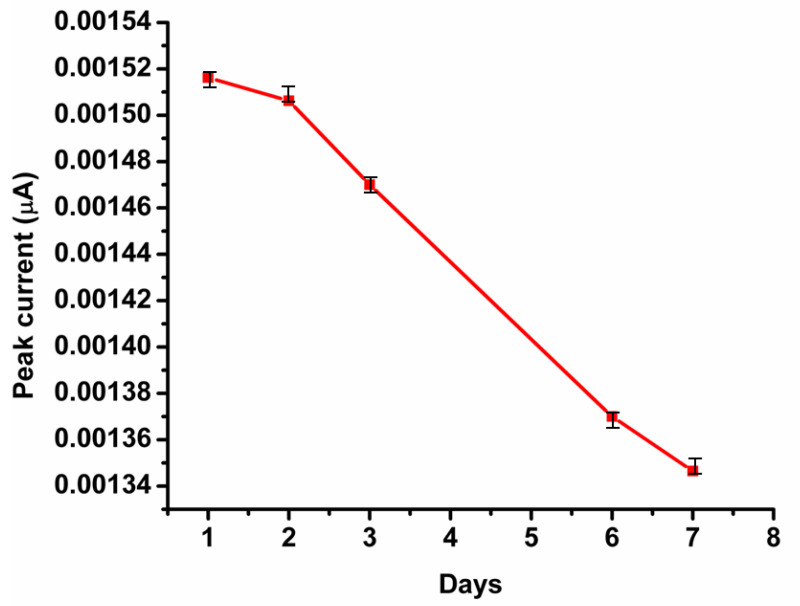
Stability of the TPP/AuNPs-S-Gr sensor for a period of 7 days.

**Figure 11:**
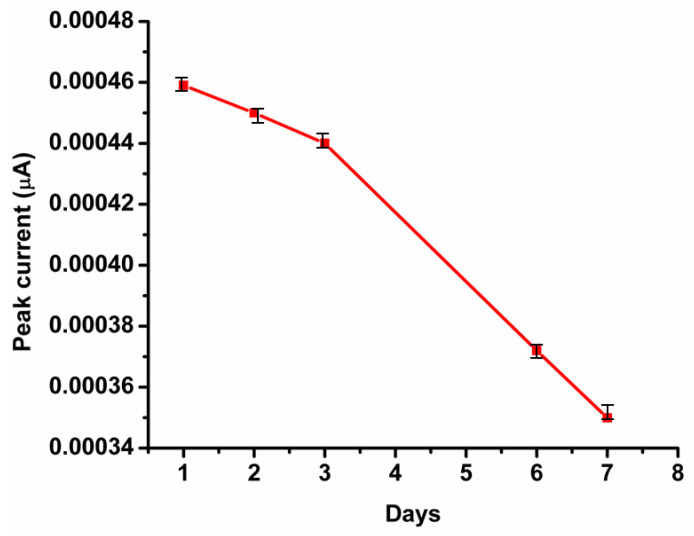
Stability of the Fe(TPFPP)Cl/AuNPs-S-Gr sensor for a period of 7 days.

The stability of the sensors was examined for 7 days. The modified electrodes were kept at room temperature throughout the stability examination. It can be observed that after 7 days, in the case of the TPP/AuNPs-S-Gr sensor (Figure 10), the current intensity of the R-LIM decreased to a value of 88.80% ± 0.94% of the initial value from the first day of assessment; and in the case of the Fe(TPFPP)Cl/AuNPs-S-Gr sensor (Figure 11), the peak current decreased up to a value of 76.23% ± 1.74% from the initial value from the first day of assessment. After the 7 day period, no significant changes in the intensity of current were observed.

Comparison between results obtained using the proposed method and those obtained using chromatographic methods is shown in Table 4.

As can be seen, the proposed method is working for higher concentrations of limonene, and wider linear concentration ranges were obtained. The advantage of the proposed sensors versus chromatographic techniques are: no dilution of the beverage sample will be needed, because the concentration of R(+)-limonene is within the linear concentration ranges; the method proposed is cost effective, and minimum pre-treatment (buffering) of samples is needed.

### 3.6. Determination of R-Limonene in Beverages

It is very important to validate the proposed sensors because their effectiveness is essential for their subsequent use in food quality control and food safety at the same time. We used the standard addition method for both sensors to validate them and to demonstrate that they are very reliable for R-LIM analysis in alcoholic beverages, such as limoncello and triple sec liqueur. We prepared the samples as shown above and for each of the samples, the R-LIM concentration was determined. After this step, known amounts of R-LIM were added, followed by measurements of its concentration. The results presented in Table 5 showed high reliability of the R-LIM test in limoncello and triple sec liqueur. When we used the TPP/AuNPs-S-Gr sensor, the recoveries were approximately 99.00% for both samples with RSD values between 0.42–2.47%. When we used the Fe(TPFPP)Cl/AuNPs-S-Gr sensor, the recoveries were greater than 99.00% for both samples, with a maximum RSD value of 2.90% for the limoncello sample and a maximum RSD value of 4.12% in the case of the triple sec liqueur sample (Table 6). Consequently, the proposed sensors can be used for R-LIM analysis in limoncello and triple sec liqueur. Sensors based on sulfur-doped graphene paste and gold nanoparticles modified with 5,10,15,20-tetraphenyl 21H,23H-porphine and 5,10,15,20-tetrakis (pentafluorophenyl)-21H,23H porphyrin iron (III) chlorides, have shown an increase the selectivity for the detection of R-LIM in alcoholic beverages; further, the recorded recoveries were high and with low RSD values (%).

Recoveries of R-limonen in the presence of S-limonen were performed to prove the enantioselectivity of the proposed sensors; results are shown in Table 7.

Results in Table 7 show that R-limonene can be determined in the presence of S-limonene despite the ratio between the two enantiomers.

## 4. Conclusions

This paper reported for the first time two electrochemical sensors based on sulfur-doped graphene (S-Gr) and a gold nanoparticle paste modified with 5,10,15,20-tetraphenyl-21H,23H-porphine (TPP) and 5,10,20,20-tetrakis (pentafluorophenyl)-21H,23H-porphyrin iron (III) chloride [Fe(TPFPP)Cl]. The proposed sensors were successfully applied in alcoholic beverages. Following the results, high stability, sensitivity, selectivity and reproducibility were recorded for both sensors. The characteristic of the proposed sensors is their use in the food industry and supermarkets for food quality control, in terms of R-LIM determination.

## Figures and Tables

**Table 1:** Response characteristics of the electrochemical sensors used for the detection of R-limonene.

Parameter	Electrochemical Sensors	
	TPP/AuNPs-S-Gr	Fe(TPFPP)Cl/AuNPs-S-Gr
Equation of calibration *	Ip_a_ = 0.0014(±0.0003) + 0.7068 × C; R^2^ = 0.9966	Ip_a_ = 0.0002(±0.0001) + 0.5027 × C; R^2^ = 0.9983
Linear concentration range (mol L^−1^)	1 × 10^−5^–6 × 10^−4^	1 × 10^−5^–1 × 10^−3^
Sensitivity (µA/mol L^−1^)	0.7068 ± 0.0020	0.5027 ± 0.0014
Limit of detection (mol L^−1^)	3 × 10^−6^	3 × 10^−6^
Limit of quantification (mol L^−1^)	1 × 10^−5^	1 × 10^−5^
Repetability of peak current (%, RSD)	2.48	3.24

* <Ip_a_> = µA; <C> = mol L^−1^, n = 10.

**Table 2:** The effect of coexisting species on the detection of 1 × 10^−4^ R-limonene (n = 5) using the TPP/AuNPs-S-Gr sensor.

Coexisting Species	Concentration of Coexisting Species (mol L^−1^)	Tolerance Limit	Relative Error (%)	Bias (%)
Mg^2+^	1 × 10^−3^	10	−1.15	0.17
K^+^	1 × 10^−3^	10	−2.51	3.94
Na^+^	1 × 10^−3^	10	−1.20	1.44
Fe^3+^	1 × 10^−3^	10	0.18	−2.03
Ascorbic acid	1 × 10^−3^	10	−1.16	−2.77
Glucose	1 × 10^−3^	10	0.78	0.10
Sucrose	1 × 10^−3^	10	0.57	−0.39
S-limonene	1 × 10^−3^	100	0.20	−0.07

**Table 3:** The effect of coexisting species on the detection of 1 × 10^−4^ R-limonene (n = 5) using the Fe(TPFPP)Cl/AuNPs-S-Gr sensor.

Coexisting Species	Concentration of Coexisting Species (mol L^−1^)	Tolerance Limit	Relative Error (%)	Bias (%)
Mg^2+^	1 × 10^−3^	10	3.45	−6.67
K^+^	1 × 10^−3^	10	4.00	−2.31
Na^+^	1 × 10^−3^	10	3.17	−5.00
Fe^3+^	1 × 10^−3^	10	−4.17	4.35
Ascorbic acid	1 × 10^−3^	10	0.99	1.00
Glucose	1 × 10^−3^	10	−3.33	−0.86
Sucrose	1 × 10^−3^	10	2.56	−0.50
S-limonene	1 × 10^−3^	100	1.23	−0.12

**Table 4:** Comparison between performances of chromatographic methods and the proposed electrochemical method.

Method	Linear Concentration Range	Limit of Detection	Reference
HPLC/MS	0.10–0.75 pg mL^−1^	0.06 pg mL^−1^	[8]
GC/MS	3.00–20.00 ng mL^−1^	1.70 ng mL^−1^	[28]
Electroanalysis using Fe(TPFPP)Cl/AuNPs-S-Gr sensor	1.94 μg mL^−1^–1.94 mg mL^−1^	580 ng mL^−1^	This work
Electroanalysis using TPP/AuNPs-S-Gr sensor	1.94 μg mL^−1^–1.17 mg mL^−1^	580 ng mL^−1^	This work

**Table 5:** The effect of coexisting species on the detection of 1 × 10^−4^ R-limonene (n = 5) using the TPP/AuNPs-S-Gr sensor.

Samples	Amount Added (mol L^−1^)	Amount Found (mol L^−1^)	Recovery (%)	RSD%
Limoncello	-	2.83 × 10^−5^	-	1.21
	1 × 10^−5^	9.92 × 10^−6^	99.20	2.33
	1 × 10^−4^	9.92 × 10^−5^	99.23	1.34
	2 × 10^−4^	1.98 × 10^−4^	99.14	1.87
Triple sec liqueur	-	1.41 × 10^−5^	-	2.47
	1 × 10^−5^	9.90 × 10^−6^	99.01	0.42
	1 × 10^−4^	9.91 × 10^−5^	99.15	0.71
	2 × 10^−4^	1.99 × 10^−4^	99.34	1.61

**Table 6:** Detection of R-Limonene in limoncello and liqueur triple sec samples using the Fe(TPFPP)Cl/AuNPs-S-Gr sensor.

Samples	Amount Added (mol L^−1^)	Amount Found (mol L^−1^)	Recovery (%)	RSD%
Limoncello	-	1.71 × 10^−5^	-	1.95
	1 × 10^−5^	9.92 × 10^−6^	99.20	0.56
	1 × 10^−4^	9.94 × 10^−5^	99.38	2.90
	2 × 10^−4^	2.00 × 10^−4^	99.78	3.08
Triple sec liqueur	-	4.24 × 10^−5^	-	0.77
	1 × 10^−5^	9.99 × 10^−6^	99.90	2.25
	1 × 10^−4^	9.93 × 10^−5^	99.31	4.12
	2 × 10^−4^	2.00 × 10^−4^	99.84	0.51

**Table 7:** Recovery of R-limonene in the presence of S-limonene (n = 10).

Sensor	S-Limonene, % Recovery			
	R:S, mol:mol			
	2:1	1:1	1:2	1:100
TPP/AuNPs-S-Gr	99.31 ± 0.05	99.87 ± 0.03	99.30 ± 0.02	99.43 ± 0.03
Fe(TPFPP)Cl/AuNPs-S-Gr	99.48 ± 0.07	99.21 ± 0.05	99.50 ± 0.06	99.47 ± 0.05

## Data Availability

Not applicable.
